# A Narrative Review on In-Hospital Alarm Fatigue and Telemetry Monitoring Failure: Epidemiology and a Safer Telemetry Framework Model Proposal

**DOI:** 10.3390/healthcare14121773

**Published:** 2026-06-19

**Authors:** Joel Shah, Sidhartha Senapati

**Affiliations:** Department of Internal Medicine, Texas Tech University Health Sciences Center, El Paso, TX 79905, USA

**Keywords:** alarm fatigue, telemetry, cardiac monitoring, patient safety, Safer Telemetry Architecture (STA), alarm management

## Abstract

**Background**: Cardiac telemetry monitoring represents an important aspect of in-hospital patient safety in both telemetry and critical care settings. Despite technological advancements, telemetry effectiveness may be diminished due to systemic failures including operational processes, instructional policies, and human factors. Alarm fatigue, recognized by the Joint Commission as a leading contributor to serious patient harm, lies at the forefront of these failures. **Objective**: This narrative review utilized and synthesized sources indexed through PubMed, PubMed Central, MEDLINE, Web of Science, Google Scholar, Directory of Open Access Journals (DOAJ), and Scopus to illustrate the factors involved in hospital related monitoring failures. We purport that alarm fatigue and telemetry monitoring failures are the result of complex systemic failures comprising technological and human failures. Through this narrative, we propose an evidence-based framework known as the Safer Telemetry Architecture (STA) to pinpoint redundancies and promote closed-loop communication regarding alarm management. **Conclusions**: Monitored in-hospital environments represent a key area of preventable morbidity and mortality due to systemic design flaws. Our STA framework addresses such flaws via improvements in nurse-driven protocols, alarm routing, mandatory coverage standards for backup, and increased performance auditing. Systemic improvements via such a framework may represent an important institutional strategy for hospitals with cardiac monitoring, but requires further prospective validation. Managing redundancies in alerts and sounds, improving backup and nursing telemetry protocols, and promoting closed or continuous loops targeting alarm response times and telemetry utilization are key to effectively improving patient safety.

## 1. Introduction

In-hospital continuous cardiac monitoring (telemetry monitoring) is vital for a large portion of patient admissions and represents the standard of care across the United States (US) [1]. Telemetry monitoring allows for detection of potentially life-threatening arrhythmias particularly in intensive care units (ICUs), step down units, and in procedural or postprocedural settings such as the post-anesthesia care unit (PACU) [1]. By providing real-time data on heart rate and rhythm, telemetry is designed to facilitate the earliest possible recognition of acute clinical deterioration, thereby functioning as a crucial defense barrier against sudden cardiac death [2]. Despite its technological sophistication and ubiquitous presence in modern hospitals, the promise of continuous monitoring may fail to translate into continuous safety. Patient safety reports demonstrate that interruptions, equipment failures, and breakdowns in the human or operational processes surrounding telemetry significantly contribute to serious patient harm [3]. An analysis of monitoring-related incidents by Kukielka et al. found that nearly all events resulting in severe temporary harm or death involved failures in the procedural or human element of monitoring rather than in the cardiac monitoring technology itself [3].

In order to understand the persistence of alarm fatigue despite technological advancements, the sonic environment of the hospital must be taken into consideration. Clinical alarms do not operate in isolation, rather they compete for attention within a highly dynamic setting characterized by shifting cognitive demands, constant interruptions, competing clinical priorities, and variable institutional culture. This environment constitutes a complex adaptive system (CAS), a network of interdependent, co-evolving subcomponents that continuously interacts and mutually influences one another [4]. Viewing telemetry failures through the lens of a CAS may help to explain why single-intervention strategies have historically failed and why a systemwide architectural solution is necessary.

The most pervasive and complex threat to safe telemetry practice is alarm fatigue: the phenomenon whereby clinicians and staff become desensitized to the volume or frequency of non-actionable alarms, ultimately failing to respond to critical events [5]. According to The Joint Commission’s Sentinel Event Alert #50 and National Patient Safety Goals, management of medical alarms is a top priority in patient safety, with alarm fatigue serving as a major contributor to adverse events [5,6]. Centralized monitoring systems, which frequently utilize a single monitor technician or nurse for numerous patients that may span multiple units, is a primary point of failure in the existing architecture of telemetry monitoring as momentary lapses in surveillance or gaps in procedural management may lead to catastrophic clinical outcomes.

This narrative review conducts a comprehensive analysis of the epidemiology of telemetry-related harm, the human factors that govern vigilance and alarm response in critical care environments, and the people, process, technology, and policy vulnerabilities that enable critical system failure in telemetry monitoring. This review synthesized information indexed in PubMed, PubMed Central, MEDLINE, Web of Science, Google Scholar, Directory of Open Access Journals (DOAJ), and Scopus to illustrate the factors involved in hospital related monitoring failures. We hypothesize that the failures pertaining to telemetry monitoring are properties of the associated CAS rather than isolated technological or human errors, and only the implementation of a systemic architectural intervention can improve patient safety and outcomes in in-hospital monitored environments. We then propose an evidence-based Safer Telemetry Architecture (STA) as a framework for establishing redundancy and closed-loop communication in alarm management to improve patient outcomes and decrease morbidity and mortality in hospitals.

## 2. Methods

### 2.1. Study Design and Rationale

As the objective of the study was to synthesize evidence on a broad topic, identify patterns to gain thematic insights, and develop a translational framework or solution, this study was designed as a narrative review. The study design was chosen as a narrative rather than a systemic review because the objectives did not require structured eligibility criteria. Narrative reviews are best suited for multidisciplinary topics such as this (which spans the nature of human error, clinical informatics, patient safety data, and health policy) as they allow for integrative synthesis across all domains [7].

### 2.2. Sources of Information

We performed a literature search utilizing seven electronic databases including PubMed, PubMed Central, MEDLINE, Web of Science, Google Scholar, the Directory of Open Access Journals, and Scopus to synthesize data for this narrative review. Sources were analyzed from database inception through April 2025. Only publications in the English language were included in this study.

Search terms utilized free-text key words with Medical Subject Headings (MeSH) including (“alarm fatigue” OR “clinical alarm” OR “monitor alarm”) AND (“telemetry” OR “cardiac monitoring” OR “patient monitoring”) AND (“patient safety” OR “adverse events” OR “sentinel events”) AND (“ICU” OR “critical care” OR “hospital”). Additional keywords included “alarm management”, “nurse alarm”, “burnout alarm”, “telemetry overuse”, “telemetry error”, “AI alarm”, “nurse-driven telemetry protocol”, and “inpatient monitoring errors”.

### 2.3. Inclusion and Exclusion Criteria

Inclusion criteria included studies that (1) addressed inpatient clinical alarm systems, alarm fatigue, and telemetry monitoring, (2) investigated individual or systemic processes, hospital policy, or other sources of monitoring failure, (3) interventions focused on promoting improvements to alarm management or patient safety, or (4) reports of epidemiological related to cardiac monitoring adverse events. Study designs included but were not limited to meta-analyses, clinical guidelines, institutional and federal or state level government reports, accreditation body publications, and systemic reviews.

Exclusion criteria included studies that (1) only addressed ambulatory monitoring without inpatient telemetry relevance, (2) contained only abstracts with full text availability, (3) had little evidence or supporting data, or (4) had a study focused on non-cardiac alarm systems.

### 2.4. Screening

All citation titles and abstracts were independently reviewed by both authors against pre-existing eligibility criteria. A total of 247 manuscripts were initially identified via database searches. After duplicate removal (n = 38) and title or abstract screening exclusion (n = 147), 62 full length articles were deemed eligible, of which 21 were ultimately chosen for consideration as primary references for the manuscript. Among the 62 eligible full length articles, final reference selection was carried out via the following prioritization criteria applied by the authors: (1) direct relevance to the manuscript’s thematic domains (alarm fatigue epidemiology, human factors, technology gaps, and policy definitions); (2) methodological rigor with a preference for systemic reviews, meta-analyses, and guidelines documentation over single-center observational studies; (3) recency of publications, with a preference for sources published within the past 15 years unless the source represented a landmark contribution or was foundational to the field; (4) source diversity, ensuring triangulation across clinical, informatics, accreditation, and human-factors-based literature. All references were cross-checked among the authors to decrease selection bias.

### 2.5. Risk of Bias

To reduce the risk of bias, including confirmation and selection bias, we developed a search strategy with inclusion and exclusion criteria alongside triangulating findings and cross-referencing multiple independent sources across all source types (human research, epidemiological data, nursing protocols, clinical guidelines, and accreditation reports).

## 3. Epidemiology of Telemetry-Related Harm

Telemetry monitoring is among the most frequently overused resources in hospital care [8]. Studies have consistently demonstrated that a substantial proportion of monitored inpatient cases do not meet evidence-based criteria for continuous telemetry monitoring which may contribute to unnecessary monitoring that inflates non-actionable alarm rates [9]. This overuse compounds alarm fatigue by increasing the background signal burden on clinical staff without an increase in actionable clinical information thereby increasing the chances of adverse events [10].

The scale of associated harm has been well-documented by The Joint Commission database, which revealed 98 alarm-related sentinel events, 80 of which resulted in death, between 2009 and 2012 [11]. According to the US Food and Drug Administration (FDA), there were 566 alarm-related deaths from 2005 to 2010 [6]. A structured analysis of telemetry-related incidents identified that human errors, including battery failures, improper lead connections, and communication breakdowns, account for approximately 47.1% of reported events [3]. The most frequently reported serious outcome across these incidents was death, underscoring the high stakes and importance of alarm management failures [3].

## 4. Critical Care-Based Human Factors and Conditioning

The cognitive demands of critical care create an environment highly susceptible to human error. Understanding the mechanisms by which alarm fatigue degrades clinical performance is essential for designing effective countermeasures [12].

### 4.1. Alarm Fatigue and Vigilance Decrement

Alarm fatigue is defined as the desensitization that occurs when a high frequency of non-actionable (false or nuisance) alarms conditions caregivers to distrust or systematically ignore subsequent alert signals [5]. Bonafide et al. revealed that only 10–20% of clinical alarms require any clinical intervention [13]. In an ICU on average, alarms may sound hundreds of times per day, creating a constant and stressful environment prone to conditioning to those alarms and turning these alarms into “background noise”. This alarm burden leads to a notable decrease in vigilance, represented by a measurable reduction in the staff’s capacity to sustain focused attention to monitoring over time [14]. The most dangerous manifestation of alarm fatigue is not active dismissal of alarms but rather conditioned inattention, a state in which clinical decision-makers have been sensitized by thousands of prior non-actionable events to implicitly assume that any given alarm is unlikely to be life-threatening. This cognitive bias has been directly implicated in delayed responses to life threatening arrhythmias and other time-sensitive emergencies across multiple published incident analyses [3,6].

### 4.2. Situational Awareness and Mental Workload

Situational awareness (SA) defined as a perception of environmental elements, comprehension of their meaning, and projection of their future status, is a prerequisite for safe patient telemetry monitoring [14]. High mental workload systematically degrades each tier of SA through the following three well-characterized mechanisms. (1) Information Overload: Central monitoring systems commonly display dozens of parameters across multiple patients simultaneously, producing visual clutter that disrupts the monitoring outflow process and fragments attentional allocation. (2) Interruption Management: Emergency events in adjacent patient care areas can fully consume the cognitive capacity and physical presence of clinical staff, functionally rendering an entire unit’s monitoring capacity non-functional for the duration of the competing demand [12]. (3) Ambient Noise: Research has consistently demonstrated that ambient noise levels in ICUs regularly exceed established safe thresholds, contributing to cognitive stress, communication errors, and masking of critical audible alarm signals [12]. Together these factors amplify the risk of monitoring failure consistent with the aforementioned CAS framework.

### 4.3. Alarm Fatigue and Healthcare Worker Well-Being

The impact of psychological well-being on the healthcare worker is an important and critically underrecognized aspect of the alarm fatigue crisis. The redundant persistence of alarm noise in the hospital environment is a known contributor of nurse burnout, anxiety, and emotional exhaustion that has been directly linked to clinical decision-making impairment and patient safety reductions [15,16,17]. These psychological adverse effects are not simply qualitative. The quantitative effects of chronic alarm exposure stem from repeated stressor activation of the hypothalamic–pituitary–adrenal axis resulting in elevations in cortisol and catecholamine levels, executive function reductions, and increases in cognitive fatigue [16]. These effects in turn have documented in-hospital behavior consequences, such as increases in deliberate alarm silencing by clinicians and healthcare workers as a self-protective response to environmental dysregulation [15,17]. This behavior constitutes a direct path by which alarm fatigue may lead to patient harm as it removes the inherent protective function of alarms thus leading to the possibility of missed events. Analysis by Albanowski et al. revealed that alarm fatigue remained a pervasive patient safety concern over 10 years after the initial sentinel alert event report by the Joint Commission with alarm silencing and nursing burnout serving as important contributors [15]. Therefore, in order to properly address in-hospital alarm safety, healthcare worker wellbeing must be simultaneously promoted.

## 5. People and Process Gaps: The Chain of Custody Failure

A comprehensive analysis of monitoring-related adverse events consistently identifies severe gaps in the established chain of custody for patient safety monitoring. These operational vulnerabilities span staffing, role definition, handoff processes, and training infrastructure [3,18]. Table 1 summarizes the key process gaps and evidence-based countermeasures central to telemetry monitoring failures.

## 6. Technology Gaps: The Fragile Single Point of Alert

Modern telemetry systems must evolve from passive reporting tools into intelligent safety architectures. The predominant technological vulnerability in current systems is their near-complete reliance on the primary auditory and visual alarm at the central monitoring station (CTS) as the sole alert pathway. Several issues lie at the forefront of this vulnerability. (1) Lack of Redundancy: When the human responsible for the CTS is absent, unavailable, or cognitively saturated, the system fails completely. There is no secondary alert mechanism to ensure that life-critical alarm information reaches clinical decision-makers [4]. (2) Absence of Smart Escalation: Without an automated, intelligent alert routing system, critical alarms can be effectively “trapped” at an unmanned or overwhelmed central station, with no time-based escalation to alternative responders [18]. (3) Alarm Parameter Defaults: Factory-default alarm settings are frequently not calibrated to the specific patient acuity or unit characteristics, generating disproportionately high rates of nuisance alarms and accelerating alarm fatigue [13].

## 7. Deficiencies in Policy

Policy governs standards and operations that dictate institutional safety. Several recurrent policy deficiencies have been identified across institutions experiencing alarm-related adverse events [21]. (1) Staffing & Coverage Standards: The absence of minimum staffing requirements and formal coverage standards for the CTS represents a fundamental policy gap that leaves monitoring vulnerable to predictable human resource constraints [3]. (2) Lack of Auditing: Failure to routinely audit and track critical performance metrics, such as alarm response time and telemetry utilization rates, ensures that systemic weaknesses persist undetected until a sentinel event forces institutional attention [1,14]. (3) Clinical Protocol Drift: Absence of active telemetry stewardship policies allows monitoring to continue well beyond evidence-based indications, directly contributing to high nuisance alarm rates and accelerating alarm fatigue at the unit level [9].

## 8. Toward a Safer Telemetry Architecture (STA): Evidence-Based Solutions

Mitigating the risks inherent in current telemetry systems requires a paradigm shift: from reactive alarm reduction strategies directed at individual behavior to the proactive construction of a Safer Telemetry Architecture (STA). Table 2 summarizes the current systemic vulnerabilities in telemetry monitoring, which the STA seeks to remedy. This architecture must guarantee redundancy and closed-loop communication as design requirements, not afterthoughts. While individual components of the STA—such as alarm middleware, nurse-driven protocols, and escalation pathways—have appeared in prior alarm management literature, the STA framework proposed here represents a novel integration of these elements into a unified, closed-loop systems architecture explicitly grounded in the complex adaptive systems (CAS) framework. Unlike previous approaches, which have tended to address single domains (technology, staffing, or policy) in isolation, the STA is distinguished by its simultaneous and interdependent treatment of technology, people, process, and policy vulnerabilities, and by its incorporation of a Continuous Improvement Feedback Loop (C-I FBL) as a first-class architectural requirement rather than an optional quality-improvement add-on. It is important to note that the STA is a conceptual framework derived from synthesis of existing evidence; it has not yet been empirically validated as a complete system, and its proposed benefits should be understood as evidence-informed hypotheses requiring prospective evaluation.

Alongside its potential value, implementation of the STA is likely to face substantial real-world barriers that must be acknowledged. From a staffing standpoint, the designation of dedicated backup monitors and enforcement of zero-tolerance CTS coverage standards may prove difficult in resource-constrained environments where nursing-to-patient ratios are already stretched. Institutions with limited capital budgets may face significant upfront costs in deploying alert router middleware and bi-directional communication infrastructure. Workflow burden represents another concern: nurse-driven telemetry discontinuation protocols introduce additional documentation and decision-making tasks into already high-demand clinical workflows, which may reduce uptake without adequate training and normative support. Interoperability challenges between legacy electronic health record systems and new alarm middleware platforms can impede seamless integration, particularly in hospitals with heterogeneous vendor environments. Finally, the customization of alarm parameters—while effective in reducing nuisance alarms—carries a risk of inadvertent under-alarming if thresholds are set too conservatively, potentially increasing the risk of missed clinical events. Future implementation research should prospectively evaluate these barriers and identify context-specific solutions to support equitable adoption across diverse institutional settings.

### 8.1. Technology Solutions: Redundant Alert Pathways

#### 8.1.1. Alert Router/Middleware and Mobile Escalation

The most critical technological intervention is the implementation of a dedicated Alert Router/Middleware system [20]. This technology serves as an intelligent gatekeeper that triages all alarms and routes only life-critical events (e.g., ventricular fibrillation, asystole, sustained ventricular tachycardia/Torsades de Pointes) directly to the mobile devices of assigned clinical staff via a predetermined, timed escalation protocol [20].

Evidence supports the superiority of bi-directional over unidirectional communication in alarm response. Studies demonstrate that replacing one-way pager-based systems with bi-directional voice or text communication devices significantly shortens time-to-first contact and dramatically increases the rate of communication loop closure from as low as 19% to as high as 100% in controlled evaluations [18].

#### 8.1.2. Alarm Prioritization and Tuning

To systematically combat nuisance alarms, often arising from premature ventricular contractions, motion artifact, or technical issues, institutions must pursue evidence-based alarm customization:

- Standardize alarm settings to adhere to AHA guidelines and customize defaults for specific unit types (e.g., CCU versus general telemetry floor) [1].
- Utilize advanced monitoring algorithms to filter artifacts and incorporate appropriate time delays before non-critical alarms are triggered, thereby reducing noise burden without compromising response to genuinely life-threatening events [13].

### 8.2. Process and Policy Interventions

#### 8.2.1. Nurse-Driven Telemetry Protocols

Institutions should adopt nurse-driven protocols (NDPs) to manage telemetry initiation and discontinuation in a structured, evidence-based manner [1]. NDPs empower bedside nurses to discontinue monitoring when AHA criteria are no longer met, significantly reducing unnecessary monitoring days and the associated alarm burden across the unit [11].

#### 8.2.2. Mandatory Backup Coverage and Escalation Protocols

Hospital policy must mandate a clear, regularly practiced Backup Coverage Protocol for the central monitoring station. This policy framework should specify:

- Immediate designation of a secondary watcher during primary staff breaks, competing emergencies, or any circumstance that would leave the CTS unattended.
- A zero-tolerance standard for unattended CTS coverage without a designated backup.
- A documented, timed escalation pathway, for example: if a critical alarm is not acknowledged at the CTS within 30 s, it must auto-escalate to the primary bedside nurse, then to a buddy nurse, then to the charge nurse, and finally to the rapid response team [18].

### 8.3. Continuous Improvement Feedback Loop (C-I FBL)

The STA framework is only as effective as its ongoing auditing and quality improvement infrastructure. A robust continuous improvement feedback loop requires:

- Performance Metrics Auditing via an analytics dashboard tracking key indicators: Critical Alarm Response Time (C-ART), time from alarm initiation to acknowledgement or intervention; Telemetry Utilization Rate, proportion of monitored patients meeting AHA indications; and Nuisance Alarm Rate (NAR), non-actionable alarms per monitored bed per day [13].
- Multidisciplinary Governance: A cross-functional team comprising nursing, clinical engineering, information technology, risk management, and physician leadership should conduct quarterly review of audit data to refine alarm parameters, update training curricula, and adjust staffing protocols [14,17,21,22].
- Proactive Risk Orientation: The goal of the C-I FBL is to move institutions beyond reacting to sentinel events toward prospective identification and mitigation of systemic vulnerabilities before they produce patient harm [1,14].

## 9. The Future of Telemetry: Remote Monitoring and Digital Health

Improving the modern-day issue of in-hospital alarm fatigue and telemetry failure requires understanding and adapting to a rapidly evolving digital health technology landscape both inside and outside the hospital setting. A meta-analysis examining digital health interventions for acute coronary syndrome revealed that implementation of a digital monitoring system improved clinical responsiveness, early deterioration detection, and overall patient adherence to care plans [23,24,25]. Additionally, artificial intelligence-based algorithms may be trained to detect subtle abnormalities in telemetry monitor heart rate variability, waveform changes, and multi-parameter signals that may be missed by current alarm systems [23]. Winters et al. highlighted that advanced algorithmic filtering of artifact and intelligent alarm prioritization represent near-term, evidence-supported strategies for reducing non-actionable alert burden in critical care settings [23,26,27]. More specifically, machine learning models trained on large inpatient arrhythmia datasets have demonstrated improved specificity in distinguishing clinically significant rhythm changes from artifact compared with conventional threshold-based alarm systems, thereby reducing false-positive alarm rates without sacrificing sensitivity to true events. Furthermore, AI-assisted predictive monitoring—where algorithms analyze trends in heart rate variability, ST-segment morphology, and multi-parameter composite scores to anticipate deterioration before it manifests as a discrete alarm—represents a promising direction supported by early feasibility studies in ICU settings, though large-scale randomized validation remains limited. The STA framework is designed to be compatible with and enhanced by such AI-based machine learning models, which may be incorporated to further reduce alarm burden and support earlier clinical intervention. As this technology matures, future updates to institutional telemetry policy and the STA framework itself should incorporate evidence-based AI tools as they achieve regulatory clearance and accumulate clinical validation data.

## 10. Conclusions

Telemetry monitoring failure resulting in preventable in-hospital morbidity and mortality is complex, with components of policy, human, and operational systems contributing to management and subsequent adverse events. Alarm fatigue, redundant alerts, and structural or transient gaps in monitoring coverage leads to a system in which a single failure, such as a momentarily unattended station or conditioning to non-response alarms, can lead to catastrophic and oftentimes fatal events.

To reduce morbidity and mortality associated with telemetry monitoring, institutional changes are necessary to shift from error response to investment into a Safer Telemetry Architecture (STA). Through heightened awareness to backup coverage tools, improvements in alarm intelligence, proper NDP updates, appropriate escalation procedures, and continued data-driven audits, hospitals and healthcare organizations as a whole may systematically and institutionally mitigate alarm fatigue thus increasing the frequency of lifesaving clinical interventions and improving continuous patient monitoring standards.

This systemic redesign is highly recommended to significantly improve patient outcomes and reduce morbidity/mortality. In high-acuity monitored environments, it constitutes an ethical and institutional obligation to every patient under continuous cardiac surveillance.

## Figures and Tables

**Table 1:** People and Process Gaps and Evidence-Based Countermeasures in Central Telemetry Monitoring.

Gap Category	Failure Mode	Evidence-Based Countermeasure
CTS Staffing & Vigilance	Unattended central telemetry station (CTS) during parallel emergencies or staff breaks, with no assigned backup coverage.	Mandate a dedicated Backup Coverage Protocol for the CTS during all emergencies or breaks; implement a formal “Watcher” designation for high-risk periods [3].
Role Ambiguity	Unclear distinction between the central monitor technician’s role (monitoring/interpretation) and the bedside nurse’s role (response/intervention).	Standardize roles and responsibilities; explicitly empower and train technicians to initiate rapid escalation codes (e.g., Code ARREST) [18].
Handoffs & Verification	Patient not connected to the monitor as ordered, or monitoring discontinued in error; inadequate handoffs during unit transfer [3,19].	Implement mandatory two-person verification steps for connecting/disconnecting telemetry; integrate monitoring status into all standardized handoff communication tools [19].
Training & Competency	Inadequate staff proficiency in alarm functions, electrode placement, and troubleshooting of common artifact sources [20].	Implement continuous mandatory education focused on alarm management, rhythm interpretation, and equipment-specific training [20].

**Table 2:** Key Systemic Vulnerabilities and Corresponding Mitigation Strategies in Telemetry Monitoring.

System Domain	Core Vulnerability	Evidence-Based Mitigation Strategy	Reference
Human Factors	Alarm Fatigue/Vigilance Decrement	Standardize alarm settings; implement time delays for non-critical alarms; use advanced artifact filtering algorithms.	[4,15]
People/Process	Unattended Central Telemetry Station	Mandatory, formal Backup Coverage Protocol and “Watcher” designation during parallel emergencies and breaks.	[3,17]
Technology	Single Point of Alert Failure; No Alarm Escalation	Implement Alert Router/Middleware for automated, bi-directional, timed mobile escalation of life-critical alarms.	[17,20]
Policy	Missing Minimum Staffing and Coverage Standards; Absence of Auditing	Policy mandate for Nurse-Driven Protocols (NDPs), minimum CTS staffing standards, and routine auditing of Critical Alarm Response Time (C-ART) and Nuisance Alarm Rate (NAR).	[1,11,14]

## Data Availability

No new data were created or analyzed in this study.
